# Mask wearing in Japanese and French nursery schools: The perceived impact of masks on communication

**DOI:** 10.3389/fpsyg.2022.874264

**Published:** 2022-11-07

**Authors:** Cécile Crimon, Monica Barbir, Hiromichi Hagihara, Emma de Araujo, Sachiko Nozawa, Yuta Shinya, Nawal Abboub, Sho Tsuji

**Affiliations:** ^1^Laboratoire de Sciences Cognitives et Psycholinguistique, EHESS, CNRS, ENS, PSL University, Paris, France; ^2^Université de Paris, Paris, France; ^3^International Research Center for Neurointelligence, UTIAS, The University of Tokyo, Tokyo, Japan; ^4^Japan Society for the Promotion of Science, Tokyo, Japan; ^5^Institute for AI and Beyond, The University of Tokyo and SoftBank, Tokyo, Japan; ^6^Institut de Psychologie, Université de Paris, Paris, France; ^7^Graduate School of Education, The University of Tokyo, Tokyo, Japan; ^8^Rising Up, Paris, France

**Keywords:** face mask, nursery school, early childhood educator, language input, child development, cross-cultural difference, online survey, communication

## Abstract

Due to the global COVID-19 pandemic, covering the mouth region with a face mask became pervasive in many regions of the world, potentially impacting how people communicate with and around children. To explore the characteristics of this masked communication, we asked nursery school educators, who have been at the forefront of daily masked interaction with children, about their perception of daily communicative interactions while wearing a mask in an online survey. We collected data from French and Japanese nursery school educators to gain an understanding of commonalities and differences in communicative behavior with face masks given documented cultural differences in pre-pandemic mask wearing habits, face scanning patterns, and communicative behavior. Participants (177 French and 138 Japanese educators) reported a perceived change in their own communicative behavior while wearing a mask, with decreases in language quantity and increases in language quality and non-verbal cues. Comparable changes in their team members’ and children’s communicative behaviors were also reported. Moreover, our results suggest that these changes in educators’ communicative behaviors are linked to their attitudes toward mask wearing and their potential difficulty in communicating following its use. These findings shed light on the impact of pandemic-induced mask wearing on children’s daily communicative environment.

## Introduction

The global COVID-19 pandemic has resulted in the pervasive wearing of face masks covering the mouth region, potentially affecting human communication at large and child language development in particular. One often mentioned way in which mask wearing might impact language development is the change in and potential degradation of auditory and visual cues. Indeed, face masks could decrease speech intelligibility (e.g., Magee et al., 2020; Smiljanic et al., 2021), or reduce facial cues relevant for language comprehension (Sumby and Pollack, 1954; McGurk and MacDonald, 1976).

However, the potential impact of mask wearing might go beyond such direct “masking” effects, where the listener’s sensory input is altered merely by virtue of a mask being worn, even if the sensory output of the communication partner is the same. Indeed, wearing a mask might also change a communication partner’s sensory output itself. For instance, adults might reduce their amount of speech due to their own discomfort while wearing a face mask, or they might attempt to talk more and articulate more clearly in order to compensate for perceived negative effects of wearing a mask. Capturing the potential effects on the quality and quantity of children’s communicative input are essential for gaining a comprehensive picture of how masked language input may affect children’s language acquisition, since both input quantity and quality are major predictors of children’s later language skills (Hoff, 2003, 2006; Huttenlocher et al., 2010; Rodriguez and Tamis-LeMonda, 2011; Weisleder and Fernald, 2013; Ramírez-Esparza et al., 2014; Hirsh-Pasek et al., 2015). The goal of the present study was to gain an understanding of the ways in which daily mask wearing might change how adults communicate with and around children.

We chose to turn to daycares as a place where children spend an important part of their days, and where mask wearing of adult daycare personnel has been the norm across the world, and was in many cases mandatory. Early Childhood Educators have thus been at the forefront of any changes in communication with children while wearing face masks.

We asked educators to compare their perception of communicative behavior in the daycare when wearing or not wearing face masks. We focused on changes in three aspects of communication that have been shown to have an effect on language acquisition: quantity of verbal communication, quality of verbal communication, and non-verbal communication. The quantity, or total amount, of speech children hear correlates with vocabulary size, language processing speed, and the ease with which they can learn new words: the greater the amount of speech a child hears, the higher her vocabulary will be (Hoff, 2003, 2006; Weisleder and Fernald, 2013). Likewise, the quality, or acoustic features, of speech can help a child learn language, through for example infant directed speech (IDS; Golinkoff et al., 2015). Finally, non-verbal communication also plays a pivotal role in language acquisition: for example, pointing or eye-gaze help children figure out the meanings of words (Brooks and Meltzoff, 2005). The amount of joint attention or the responsiveness of the child’s interlocutor toward them are also correlated to their vocabulary growth (Pan et al., 2005; Huttenlocher et al., 2010; Rodriguez and Tamis-LeMonda, 2011; Topping et al., 2013; Ramírez-Esparza et al., 2014; Hirsh-Pasek et al., 2015; Smith et al., 2018). Through a series of statistical analyses, we then aimed to characterize the interaction between these factors.

To get a clearer picture of the perceived effect of masks, we conducted our survey with early childhood educators in two countries: France and Japan. While these two countries are comparable in their early childhood care systems and mask use during the pandemic, the variables that differed were linguistic and cultural. French and Japanese have different sound inventories that may have been affected differently by mask wearing (Nguyen et al., 2021a). On a similar note, mask wearing practice was different in France and Japan before the pandemic: in Japan it was common in flu and allergy season, but mostly uncommon in France (Saadatian-Elahi et al., 2010; Nakayachi et al., 2020). These factors may influence the perceived effect of masks, so any common trends would be indicative of broadly generalizable effects.

### Potential negative impact of facemasks on communicative behavior in daycares

Wearing a face mask might affect a speaker’s and a listener’s communicative behavior. For instance, the discomfort of wearing a face mask, or beliefs about the effects of masked communication, might impact the frequency and manner in which educators speak. Previous literature suggests that educators’ speech can be affected by environmental and internal factors. For example, the speaker’s mood can impact their speech rate (Ellgring and Scherer, 1996), articulation rate or average syllable duration (Alghowinem et al., 2012), while F0 and sound pressure level vary depending on the time of day, associated to vocal fatigue (Jónsdottir et al., 2002; Laukkanen and Kankare, 2006). In fact, several studies have reported discomfort caused by wearing a mask (Shenal et al., 2012; da Cunha-Martins et al., 2021). In Ribeiro et al. (2020), participants having to wear a mask while working reported increased difficulty in coordinating speech and breathing, vocal fatigue and avoidance of voice use compared to participants wearing masks only to perform sporadic essential activities. Thus, it is conceivable that prolonged mask wearing could alter the way educators speak by causing discomfort (but see Mitsven et al., 2022). Such a change in children’s language input could potentially have important consequences on their language development, as quantity and quality of speech and non-verbal communication are important predictors of young children’s language skills.

Moreover, communication is not a one-way street, and children adjust their own speech production based on the input they receive (e.g., Goldstein and Schwade, 2008). Thus, changes in educators’ communication might affect the way children listeners themselves communicate, leading to cascading effects on mutual communication, in turn affecting the educators’ speech again. In addition, such effects could occur both regarding direct input to the child and overheard communication from interactions between educators. However, the little existing evidence that looked at the impact of children themselves wearing a mask in educational settings during COVID suggest that mask wearing might not impact children’s speech that much (Mitsven et al., 2022), and thus, the jury is still out on to what extent children contribute to changed communication patterns during one-way or mutual mask wearing.

### Potential compensatory communicative mechanisms while wearing face masks in daycares

So far, we have focused on potential negative impacts of wearing masks on communication through changes in the way educators talk. It is, however, also possible that such negative effects are compensated for by other means.

Speakers have indeed been shown to be able to modify their speech quality (speech rate, amplitude, pitch, length of words, distance between contrasting categories), to compensate for difficult communication situations. This can be found in Lombard speech, naturally occurring in noisy environments (Brumm and Zollinger, 2011), or in the case of clear speech style, where the speaker pays particular attention to their language production, for instance when interacting with a hearing-impaired interlocutor (Krause and Braida, 2004). Both of these compensatory mechanisms have been shown to increase intelligibility for the listener (Picheny et al., 1985; Uchanski et al., 1996; Pittman and Wiley, 2001), and could alleviate masks’ potential impact on intelligibility (Smiljanic et al., 2021).

In addition to adaptation of speech quality, non-verbal communicative cues could help language processing. While articulatory cues might be made unavailable in the context of mask-wearing, other body movements are readily accessible. For instance, naturally occurring iconic co-speech gestures can be successfully used by listeners to compensate for speech degradation (Obermeier et al., 2012; Drijvers and Özyürek, 2017; Drijvers et al., 2019). Similarly, the repetitive movements or beat gestures from the head, hands or eyebrows have been shown to correlate with speech characteristics such as pitch accent, prosodic phrases and even discourse structure (Graf et al., 2002; Flecha-García, 2010; Swerts and Krahmer, 2010; Esteve-Gibert and Prieto, 2013), and are integrated early on during language processing (Biau and Soto-Faraco, 2013; Wang and Chu, 2013). In the absence of other cues, it remains to be studied how these movements could help language processing and whether those cues could be available to children and young infants. Finally, speakers’ eye gaze is linked to the content of the utterance (Griffin and Block, 2000), and has been shown to contribute to language comprehension (Knoeferle and Kreysa, 2012; Sekicki and Staudte, 2018), and disambiguation (Hanna and Brennan, 2007). Moreover, eye gaze is an example of how listeners can, just as speakers, adapt to the quality of the input. Indeed, listeners’ reliance on speakers’ gaze cues has been shown to increase with ambiguity (Macdonald and Tatler, 2013). This could also prove to be the case in young children as they have been shown to increase fixations to the speaker’s face in noisy environments (Macdonald et al., 2020).

### Degradation of the auditory and visual linguistic input while wearing face masks

A further way in which masks could affect communication is by altering the way the auditory and visual signal is transmitted to children. Early on, infants show a sensitivity to the audio-visual temporal synchrony in the speech signal (Lewkowicz, 2010), and can use this cue to detect and discriminate syllables in noise as early as 6 months of age (Lalonde and Werner, 2019). In addition to temporal visual cues, children this age can use articulatory cues for better phonemic discrimination, and their performance increases when the auditory input is accompanied by the corresponding visual stimulus (Teinonen et al., 2008).

Because they obstruct the airflow as it comes out of the mouth of the speaker, and hide the mouth region, face masks impact both auditory and visual speech cues at the same time (see Nguyen et al., 2021a,b, for spectral analyses of speech with and without a surgical mask, and Magee et al., 2020, for an acoustic comparison between mask types). The question remains on how adults and children can accommodate this type of altered linguistic input.

The little research that came prior to the pandemic had found no added cost in speech perception, for normal hearing adults listening to speech through a mask (Mendel et al., 2008; Atcherson et al., 2017). But several recent papers have reported decreases in speech perception in mask contexts (Radonovich et al., 2009; Yi et al., 2021), due to their impact on auditory (Rahne et al., 2021), or visual cues (Haider et al., 2022). Interestingly, some of those new laboratory studies presented more ecological conditions (e.g., conversational speech stimuli or the use of human speech as noise). In a survey in healthcare settings, Lee et al. (2022) showed an increase in listening effort and cognitive load in mask-wearing contexts. These recent results highlight the need for studies on the impact of masks on speech perception in naturalistic settings.

Regarding children, the research is still in its infancy. However, in a looking preference paradigm comparing 2 and 3-year-olds’ ability to recognize familiar spoken words, Singh et al. (2021) and Singh and Quinn (2022) found no difference between a no mask and an opaque mask condition, suggesting young children are capable of adapting to the absence of visual cues from the lower face.

School environments are notoriously loud, with high levels of ambient irregular noise coming from conversations, vocalizations and toys (see Picard, 2004; Yassin et al., 2016; Royo-Llonch, 2020). Even outside of a mask wearing context, the ambient noise demands adjustments to the communicative behavior, and daycare educators report the need to frequently repeat themselves or raise their voice (Pope, 2018). Moreover, this ambient noise specifically impacts young children, as they show greater difficulty than adults for language processing in noisy environments (Newman, 2009; Leibold et al., 2016; Oster and Werner, 2017). In these conditions, the potential effects of masks could be heightened, both for adults and children. In this paper, we will focus on the potential alterations to the communicative behavior of speakers and listeners, how they might adjust other aspects of their behavior to compensate for those changes. To that end, we constructed an online survey to gather Early Childhood Educators’ perceptions of their own communicative behavior (verbal and nonverbal), as well as the communicative behavior of their interlocutors (other masked educators and children), in mask wearing contexts. To explore which type of behaviors might be impacted and how, we assessed their perceptions relating to their language quantity and quality (e.g., speech volume and articulation quality), as well as non-verbal communicative behaviors (e.g., gestures or facial expressions), as all of these have been shown to play a role in language development. Looking for other factors that could modulate those behaviors, we also gathered information on the perceived difficulty of communicating with masked interlocutors for the surveyed educators themselves, as well as for their communication partners. Similarly, we asked about educators’ attitudes toward mask wearing and their knowledge of language development processes.

### Previous facemask experiences and cultural differences

Before reaching school age, an important proportion of children is cared for in a structure outside of the home, where they can spend the majority of their awake time. In France, this concerns about 45% of the children from 0 to 3 years of age, with 13% registered in a daycare as the primary care system (Observatoire National de la Petite Enfance, 2021). In Japan, 40.3% of children under 3 years of age attend daycares (divided between *hoikusho* and *kodomoen*, two types of structures that both accommodate children from 0 to 5; Ministry of Health, Labour and Welfare, 2021).

While in both France and Japan, mask wearing policies or recommendations during the pandemic involved wearing a mask at all times in public spaces and at work, the experience people had with mask wearing prior to the pandemic differed. In Japan, the habit of wearing a mask was widespread before the COVID-19 pandemic (Chiyoma, 2019; Miyazaki et al., 2021; Sakakibara and Ozono, 2021), and face masks were considered a daily life item. Despite just being encouraged to do so, more than 80% of Japanese people wore a mask during the pandemic (Ministry of Health, Labour and Welfare, 2020), and started doing so early on in the pandemic (Nakayachi et al., 2020). In France, people did not use facemasks in their daily lives prior to the pandemic (Saadatian-Elahi et al., 2010), and it was largely restricted to sales in pharmacies. Its use was mainly limited to medical situations (for example at the hospital), or in situations of exposure to chemicals or dangerous agents (for example in the context of construction works). This difference in prior experience with mask wearing might allow us to explore experience’s role in the perception educators have of the impact of facemasks on communication.

### Research questions

Firstly, to explore the potential impact of masks on communicative behaviors, we aimed to understand whether educators in both countries report a change in their own, other educators’, and children’s communicative behaviors, when comparing masked and unmasked contexts. To this end, we constructed subscales of questions capturing educators’ own (Subscales I–III in Methods) and others’ (Subscales V-VI) perceived verbal and non-verbal communication. To capture perceived changes in ease of communication, we asked about perceived changes in educators’ own (Subscale IV) and others’ (Subscale VII) perceived ease of communication.

Secondly, given such changes were reported, in order to get a better understanding of how masks’ impact unfolds, we explored how some factors, internal or external, might be linked with those potential changes. To study potential dependencies, including compensatory mechanisms, between interlocutors’ communicative behaviors, we looked at the relationship between participants’ reported communicative behavior and their interlocutor’s behavior (whether it be a colleague or a child), when they have to interact using a face mask (see Question 1 in the Results section). To explore the existence of dependencies, including compensatory mechanisms, within a single speaker, we will look at the dynamics between the different subtypes of communicative behaviors within the educators themselves: e.g., does a change in the speaker’s perceived language quantity correlate with a change in non-verbal communicative behavior such as gestures (Questions 2 and 3)? Finally, we will look at how internal factors might modulate those reported changes. We will consider how attitudes toward mask wearing impact the perceived difficulty of exchange, and how this difficulty relates to perceived changes in communicative behavior of the participants and their colleagues (Questions 4, 5, and 6). Lastly, we question whether participants’ knowledge of language development relates to their level of worry regarding children’s language development in the context of mask wearing (Question 7).

For all these factors, we will also include country as a predictor to examine potential differences between educators in France and Japan.

## Materials and methods

### Study design

We conducted a pre-registered online survey for daycare staff in France and Japan on Qualtrics^1^ in the summer of 2021. The survey was designed to take approximately 15 min to answer. We focused on staff who looked after young children under 3 years of age (i.e., infants and toddlers). We tested educators from two daycare groups, one in Japan and one in France, which were chosen to match on the following criteria. They both were large daycare groups with more than 100 daycares; they were private daycare groups that received financial support from the government; and they were daycares located in areas with a wide distribution of average socioeconomic status. The questions for the online survey were constructed by the authors using an iterative process, in consultation with representatives of both daycare groups, and until all authors unanimously agreed. This study was approved by the Office for Life Science Research Ethics and Safety, The University of Tokyo. The preregistration document and questionnaire items are available in the open science framework (OSF) project repository.^2^

### Participants

Each daycare director was sent an email, containing a rationale for the survey, and the link to complete the online survey. They were then in charge of disseminating this email to their employees. There was no compensation for participating in the survey.

The French sample was collected between July 13th and August 15th, 2021. Of the 383 accesses in total, 177 participants were included in the final analysis. Exclusion reasons were as follows: accessed the welcome page of the survey but did not respond (*n* = 118); stopped answering before the completion of the first five sections (survey explanation, consent form, general information, instructions, interactions with children) of the survey (*n* = 16); answered out of the data collection period (*n* = 8); not being an educator (*n* = 45); less than 2 years of experience (*n* = 19), as they would not have known what communication in daycares was like without daily mask-wearing before the pandemic. The Japanese sample was collected between July 27th and August 20th, 2021. Of the 346 total accesses, 138 participants were included in the final analysis. Exclusion reasons were as follows: only accessed the welcome page (*n* = 158); stopped answering before the completion of the first part (*n* = 16); not being an educator (*n* = 16); less than 2 years of experience (*n* = 18). Thus, we obtained a total of 315 participants in this study. The sample characteristics are shown in Table 1.

**Table 1:** Sample characteristics by country.

	French (*n* = 177)	Japanese (*n* = 138)
**Age**	35.6 (8.5)	36.9 (11.8)
**Years of experience**	9.1 (6.5)	8.8 (6.8)
**Gender** Female Male Prefer not to answer	97.2% 1.7% 1.1%	95.7% 4.3% 0%
**Job position** Director Educator Assistant	40.7% 23.7% 35.6%	13.8% 73.2% 13.0%
**Native language**	French: 97.2%	Japanese: 100%
**Daycare area** Urban Not urban N/A*	83.1% 15.8% 1.1%	87.0% 11.6% 1.4%
**Size of daycare** Less than 20 children Between 20 and 40 children More than 40 children N/A*	11.9% 44.1% 42.9% 1.1%	10.9% 12.3% 74.6% 2.2%
**Opportunity to interact with infants (< 12 months)** Never Sometimes Often	1.1% 24.3% 74.6%	21.0% 44.9% 34.1%
**Opportunity to interact with toddlers (> = 12 months)** Never Sometimes Often	0.0% 13.6% 86.4	2.1% 22.5% 75.4

^*^Either not answered or answered as more than one option (e.g., checked both ‘Urban’ and ‘Not urban’). For participants’ age and years of experience, means (SDs) are shown.

### Survey construction

We constructed the survey such that participants were asked to report whether they perceived any differences between when they wore a mask and when they did not. The items for Subscales I to VII were constructed as six-alternative forced-choice questions, consisting of five options equivalent to ‘more negative’, ‘a little more negative’, ‘no-change’, ‘a little more positive,’ and ‘more positive’ with an additional ‘I do not know’ option. For instance, the question ‘When I wear a mask with the children, I feel like I’m talking…’ had answering options of ‘much less,’ ‘a little less,’ ‘just as much,’ ‘a little more,’ ‘much more,’ and ‘I do not know’.

The questions in Subscales I–IV focused on educators’ perception of their own communication with children or colleagues. The questions in Subscales V–VII addressed children’s or colleagues’ communication as perceived by the educators. For Subscales VIII–X, we asked about educators’ own worries about the impacts of mask-wearing on children’s development, their own knowledge about language development, and their attitudes toward wearing a mask, respectively.

Depending on their answer to a general question about opportunities to interact with infants or toddlers, participants answered different sets of questions in terms of children’s communication (for Subscales V–VII). For instance, if a participant answered ‘never’ to the question of a chance to interact with toddlers, the questions related to toddlers’ communicative behavior were skipped.

Note that while Subscales V–VII do differentiate between infants and toddlers, Subscales I–IV do not. Given that questions in the latter would have had identical formulations for both ages, and to avoid lengthening the survey too much, we did not separately ask for both ages. The same did not hold for the former, as depending on the age, educators were for example asked about children’s babble or sentences, and we thus asked separately for infants and toddlers.

#### Subscale I: Own language quantity

This subscale addressed the quantitative aspects of the educators’ perception of their own communicative behavior toward children or team members when conversing in the context of mask-wearing. For communication with colleagues, three individual question items addressed caregivers’ perceived amount of speech, sentence length, and length of conversation. For communication with children, an additional fourth item addressed the frequency of reading stories and singing.

#### Subscale II: Own language quality

This subscale addressed the qualitative aspect of the educators’ perception of their own communicative behavior toward children or team members with three question items: adjustment of speech volume, clarity of articulation, and repetition of utterances when wearing a mask.

#### Subscale III: Own non-verbal cues

This subscale probed the educators’ perception of their own non-verbal communicative behavior, that is, exaggerated gesture use or facial expression toward children or team members. An additional item regarding getting down at the children’s level to interact with them was included in this subscale toward children. Thus, for communication toward colleagues, there were two items, and three items for children.

#### Subscale IV: Own ease of exchange with team members

In this subscale, participants were asked to answer the extent to which they felt ease or difficulty in exchanging with colleagues who wore a mask. The three items concerned hearing what colleagues said and understanding what they said and their facial expressions.

#### Subscale V: Others’ verbal communicative behavior

This subscale addressed the participants’ perception of others’ verbal communicative behavior in the context of masked interaction with the participant. We subdivided ‘others’ into ‘infants,’ ‘toddlers,’ and ‘team members’ as verbal communicative behavior might differ between these groups. For infants, there were two items: the frequency of cooing/babbling and the frequency of repetition of what educators said. For toddlers, there were three items: the frequency of word/sentence production, the frequency of repetition of what educators said, and clearness of pronunciation. For team members, there was a single item asking about the frequency of their coming to talk to educators.

#### Subscale VI: Children’s non-verbal communicative behavior

This subcategory addressed the participants’ perception of infants’ or toddlers’ non-verbal communicative behavior toward the educators themselves while they were wearing a mask. There were five different questions with the same wording for both age groups, addressing smiling, imitation, gesture use, physical contact, and eye contact.

#### Subscale VII: Others’ ease of exchange with the educator

In this subscale, participants were asked about the extent to which they perceived that others (i.e., infants, toddlers, and team members) felt ease or difficulty exchanging with them. There were two items, addressing ease of hearing and of comprehension for each group.

#### Subscale VIII: Own worry about children’s development

Here, we asked how concerned the participants were about children’s development when all daycare staff wore a mask, with a gliding continuous scale going from ‘I am worried’ (coded 0) to ‘I am optimistic’ (coded 100). We also asked about the participants’ perception of the proportion of team members and the children’s primary caregivers they thought worried about the impact of mask-wearing on child development.

#### Subscale IX: Own language development knowledge

To assess language development knowledge, we created six items assessing participants’ general knowledge about language development during infancy and toddlerhood, based on Suskind et al. (2018). Some examples of the items and the correct answers are: ‘Being able to see people’s lips is essential for language acquisition’ (False); ‘Young children learn language through different senses (sight, smell, touch etc.)’ (True); ‘Responding to the child only when they use words, not just when they point, helps them learn to speak’ (False). For each item, there were five options: ‘do not agree at all’, ‘do not agree,’ ‘neither agree nor disagree,’ ‘agree,’ and ‘totally agree.’

#### Subscale X: Own attitude toward mask wearing

In this subsection, participants were asked about their attitudes toward wearing a mask with yes/no questions. There were four questions: (1) ‘I find wearing a mask uncomfortable;’ (2) ‘When I wear a mask, I can forget I am wearing it;’ (3) ‘I think it is useful to wear a mask at nursery schools to control the pandemic;’ (4) ‘I think masks, be it at the nursery or outside of work, are essential to control the pandemic.’ Note that the valence of item (1) is inverted with respect to the other items.

#### Other questions

In addition to the questions in the subscales, we asked other questions that we deemed important but that did not fit into any subscale. The questions included educators’ perception of the ease or difficulty with the establishment of a new relationship with children, children’s eagerness to exchange with the educator, children’s preference for the educator wearing a mask, and children’s adaptation to situations of mask-wearing. We do not report the results of these items in the main text as they are beyond the scope of our primary analyses.

### Data analysis

#### Indices

For Subscales I-VII, we converted Likert scale responses into numeric ones ranging from -2 (more negative) to 2 (more positive) so that the neutral ‘no-change’ option (corresponding to an absence of change observed), was set as 0. For instance, a 5-levels Likert scale of ‘much less,’ ‘a little less,’ ‘as much,’ ‘a little more,’ and ‘much more’ was converted to the numeric variables of −2, −1, 0, 1, 2, respectively. This conversion was executed to make the interpretation more straightforward. We then calculated the mean score per subscale for each participant. If there were ‘I do not know’ responses to an item in a given subscale for a given participant, we calculated the participant’s subscale mean score from the remaining subscale items. If there were only ‘I do not know’ responses for a participant’s subscale, we excluded the subscale for the participant.^3^

Since Subscale VIII, ‘own worry about children’s development,’ only had one item, we used that item’s response value directly. For Subscale IX, ‘own language development knowledge,’ responses to each item were coded in 5 steps. Thus, for each item, the score ranged between 0 (the most incorrect option) and 4 (the most correct option). We then summed up all the item scores for each participant. The sum score of this subscale ranged from 0 to 24 as it consisted of 6 questions. For Subscale X, ‘own attitude toward mask wearing,’ we counted the number of ‘yes’ responses, except for item (1). Since this item was inverted, 1 point was given if the answer was ‘no,’ and 0 if ‘yes.’ Thus, the sum score of this subscale ranged from 0 (more negative) to 4 (more positive).

We excluded a given subscale if it was incomplete (i.e., no response). The summary of indices calculated for each subscale is shown in Table 2.

**Table 2:** Summary of indices.

Subscale	Answer format	Score range for each item	Summary method	Score range for each subscale
I: Own language quantity II: Own language quality III: Own non-verbal cues IV: Own ease of exchange with team members V: Others’ verbal communicative behavior VI: Children’s non-verbal communicative behavior VII: Others’ ease of exchange with the educator	5-levels Likert scale	−2 to 2	Averaged	−2 to 2
VIII: Own worry about children’s development	Continuous scale	0 to 100	As it was	0 to 100
IX: Own language development knowledge	5-levels Likert scale	0 to 4	Summed	0 to 24
X: Own attitude toward mask wearing	Yes/No scale	0 or 1	Summed	0 to 4

#### Internal consistency for each subscale

To assess the internal consistency of each subscale, we calculated Cronbach’s alpha when the subscale included two or more items. We descriptively evaluated the internal consistency, and further confirmed r.drop values, the correlations between the item and the scale composed of the remaining items, for subscales with a Cronbach’s alpha of less than 0.7 (Kline, 1999). We performed statistical analyses regardless of the outcomes of this assessment. However, we explored how results changed in case we dropped certain items with non-negligible low r.drop values when, by dropping these items, alpha values increased or the difference between subscales in the analysis decreased.

#### Comparison of subscale scores against no-change and between countries

Our first set of analyses addressed to what extent participants perceived changes when comparing daycare settings in which they wore a mask to those where they did not. To that end, we compared the mean scores against the ‘no-change’ option (i.e., 0) using a one-sample *t*-test for each of the Subscales I–VII, separately for French and Japanese respondents. Holm’s method was used for value of *p* correction.

We also were interested in evaluating cultural differences, and therefore compared scores for each Subscale (I–X) between French and Japanese respondents using independent *t*-tests. Again, Holm’s method was used for adjusting value of *p*s.

#### Linear regression modeling

Our second set of analyses addressed the potential relationships between those reported communicative behaviors. To this end, we performed a series of linear regression analyses relating to the relevant subscale scores for each hypothesis. Unless specified otherwise, each of these regression analyses also includes country, and the interaction between each predictor subscale and country as independent variables.^4^

## Results

### Preprocessing and internal consistency

First of all, we assessed the internal consistency of each subscale using Cronbach’s alpha. The values varied across subscales, ranging from 0.35 to 0.85 (Table 3). Many of the subscales did not meet the rule of thumb of the acceptable internal consistency of more than 0.7 (Kline, 1999). For subscales with low internal consistency, we further confirmed r.drop values for each item, with r.drop values higher than 0.3 considered as acceptable. The items whose r.drop value was equal to or less than 0.3 included ‘sentence length’ in subscale ‘own language quantity toward children’ (r.drop = 0.29), ‘articulation’ in subscale ‘own language quality toward children’ (r.drop = 0.30), ‘smile’ and ‘eye contact’ in subscale ‘non-verbal communicative behavior in infants’ (r.drop = 0.27 and 0.23, respectively), and ‘smile,’ ‘imitation,’ ‘physical contact,’ and ‘eye contact’ in subscale ‘non-verbal communicative behavior in toddlers’ (r.drop = 0.12, 0.17, 0.25, and 0.05, respectively). Thus, although we performed the subsequent comparison and regression analyses, we want to caution the reader that those subscales might not have had sufficient validity as summarized variables, and the items should rather be regarded individually. We are, additionally, reporting whether and how results change when excluding items with low r.drop values.

**Table 3:** Means, SDs, and Cronbach’s Alpha for each subscale.

Subscale	French	Japanese	Alpha	Comparison	Research questions
**Own language quantity** Toward children Toward team members	**−0.25 (0.58)** (177) −**0.26 (0.73)** (156)	0.01 (0.35) (138) −**0.15 (0.40)** (127)	0.59 0.71	Fr < Ja n.s.	1, 2, 4, 5 1, 2, 4, 5
**Own language quality** Toward children Toward team members	**1.15 (0.63)** (177) **1.15 (0.68)** (156)	**0.62 (0.51)** (138) **0.45 (0.50)** (127)	0.56 0.69	Fr > Ja Fr > Ja	1, 2, 4, 5 1, 2, 4, 5
**Own non-verbal cues** Toward children Toward team members	**0.77 (0.61)** (177) **0.65 (0.73)** (156)	**0.64 (0.54)** (138) **0.39 (0.57)** (127)	0.50 0.48	n.s. Fr > Ja	1, 2, 4, 5 1, 2, 4, 5
**Own ease of exchange** **with team members**	**−1.17 (0.57)** (155)	**−0.65 (0.48)** (126)	0.82	Fr < Ja	4, 6
**Verbal communicative behavior** In infants In toddlers In team members	**−0.63 (0.69)** (154) −**0.46 (0.60)** (156) −**0.14 (0.48)** (152)	**−0.23 (0.52)** (92) −**0.14 (0.40)** (126) −0.02 (0.31) (124)	0.62 0.74 N/A	Fr < Ja Fr < Ja n.s.	1, 3 1, 3 1
**Non-verbal communicative behavior** In infants In toddlers	−0.09 (0.52) (157) 0.04 (0.44) (159)	−0.04 (0.38) (96) −0.01 (0.25) (128)	0.55 0.35	n.s. n.s.	1, 3 1, 3
**Ease of exchange with the educator** For infants For toddlers For team members	**−0.85 (0.61)** (155) −**1.00 (0.55)** (159) −**1.25 (0.61)** (156)	**−0.56 (0.55)** (90) −**0.54 (0.50)** (128) −**0.53 (0.52)** (125)	0.76 0.78 0.85	Fr < Ja Fr < Ja Fr < Ja	5, 6 5, 6 5, 6
**Own worry about children’s development**	43.7 (29.2) (144)	45.6 (32.6) (116)	N/A	n.s.	7
**Own language development knowledge**	16.61 (2.73) (145)	14.52 (3.32) (118)	N/A	Fr > Ja	7
**Own attitude toward mask wearing**	1.93 (1.11) (145)	2.62 (0.77) (118)	N/A	Fr < Ja	6

For subscales from ‘own language quantity’ to ‘ease of exchange with the educator,’ scores are shown in bold when they significantly differed from the ‘no-change’ option (i.e., 0). The results of the cross-cultural comparison between the countries are shown in the ‘comparison’ column. Correspondences between the research questions and subscales used for the analyses are shown in the ‘Research questions’ column. Means (SDs) are shown in the columns of French and Japanese, with the second parentheses indicating the numbers of participants.

### Subscale scores against the ‘no-change’ option

Next, we explored whether each subscale score was significantly different from the ‘no-change’ option. For many communicative behaviors perceived by the participants, the subscale scores were significantly different compared with situations where they did not wear a mask (*t* statistics, adjusted value of ps and effect sizes are shown in Supplementary Table S1). Specifically, the subscales measuring educators’ perceived own language quality and own non-verbal cues were significantly enhanced in the context of mask-wearing regardless of country and listener (*t* statistics ranged from 7.75 to 24.09, all value of *p*s were lower than 0.001, *d* ranged between 0.69 and 1.81). For the subscales measuring educators’ perceived own language quantity, however, French participants perceived to significantly reduce the amount of verbal communication toward both children (*t* = −5.77, *p* < 0.001, *d* = 0.43) and team members (*t* = −4.46, *p* < 0.001, *d* = 0.36), whereas Japanese participants perceived to significantly reduce their own language quantity toward team members (*t* = −4.34, *p* < 0.001, *d* = 0.38) but not toward children (*t* = 0.43, *p* = 1.0, *d* = 0.04).

The verbal communicative behavior of others (infants, toddlers, and team members) perceived by the participants marked significantly lower scores when the participants wore a mask than when they did not (*t* statistics ranged from −11.40 to −3.58, value of *p*s ranged between less than 0.001 and 0.0032, *d* ranged from 0.29 to 0.92; see Supplementary Table S1 for details), except for team members’ verbal communicative behavior in Japan (*t* = −0.58, *p* = 1.0, *d* = 0.05). On the other hand, neither infants’ nor toddlers’ non-verbal behavior as perceived by the participants was significantly different from the ‘no-change’ option.

Regarding own and others’ ease of exchange, all subscale scores were significantly lower than the ‘no-change’ option (*t* statistics ranged from −25.76 to −9.68, all value of *p*s were less than 0.01, *d* ranged between 1.02 and 2.06). This suggests that the participants felt that both they and others (i.e., infants, toddlers, and team members) experienced difficulty of exchange in the context of mask-wearing.

Note that these results were mostly consistent even when the item with a low r.drop value of ‘articulation’ (r.drop = 0.30) was eliminated from the subscale ‘own language quality toward children,’ and the item of ‘eye contact’ (r.drop = 0.05) was eliminated from the subscale ‘non-verbal communicative behavior in toddlers.’ One exception was that, after eliminating the item ‘eye contact,’ the subscale in French became significant (Mean = −0.12, SD = 0.47, *t =* −3.20, *p* = 0.010, *d* = 0.25), suggesting that toddlers in French daycares decreased their non-verbal communicative behavior when the educators wore a mask.

### Comparison of subscale scores between French and Japanese samples

We then asked whether each subscale score differed between the two countries. The summarized results are shown in Table 3 and the detailed results are shown in Supplementary Table S2. Own language quantity toward children was significantly lower in the French sample than the Japanese sample (*t* = −5.01, *p* < 0.001, *d* = 0.54), suggesting that French educators perceived that they reduced the amount of verbal communication toward children when wearing a mask. Meanwhile, French educators enhanced their perceived own language quality and own non-verbal cues significantly more than Japanese educators (*t* statistics ranged between 3.35 and 9.94; value of *p*s were between less than 0.001 and 0.0064; *d* ranged from 0.39 to 1.15), except for own non-verbal cues toward children (*t* = 1.90, *p* = 0.29, *d* = 0.21).

For others’ communicative behavior, French educators perceived both infants and toddlers weakened their verbal communicative behavior significantly more than Japanese educators (infants: *t* = −5.18, *p* < 0.001, *d* = 0.64; toddlers: *t* = −5.34, *p* < 0.001, *d* = 0.61), but such cultural differences were not observed in verbal communicative behavior in team members and non-verbal communicative behavior in infants/toddlers.

Regarding participants’ own and others’ ease of exchange, all subscale scores were lower in the French sample compared with the Japanese one (*t* statistics ranged from −10.80 to −3.74; value of *p*s were between less than 0.001 to 0.0019; *d* ranged between 0.48 and 1.27), suggesting that French participants felt that both they and others interacting with them had more difficulties in exchanging with each other than Japanese participants.

For the remaining subscales, participants’ own worry about children’s development did not significantly differ between countries (*t* = −0.50, *p* = 0.63, *d* = 0.06). However, French participants marked significantly higher scores on their own language development knowledge (*t* = 5.49, *p* < 0.001, *d* = 0.69). French participants had significantly more negative attitudes toward wearing a mask (*t* = −5.91, *p* < 0.001, *d* = 0.71).

Note that the results regarding the subscales ‘own language quality toward children’ and ‘non-verbal communicative behavior in toddlers’, were consistent even when the items ‘articulation’ and ‘eye contact’ were eliminated.

### Question 1: Relationship between educators’ and others’ communicative behavior

We explored whether and how educators’ perception of others’ communicative behavior (i.e., that in children and team members) correlated with their own perceived communicative behavior toward others. For the relationship between educators’ and children’s behavior, we performed three linear regression models with ‘verbal communicative behavior in infants/toddlers’ and ‘non-verbal communicative behavior in infants/toddlers’ as predictors of educators’, ‘own language quantity,’ ‘own language quality,’ and ‘own non-verbal cues’ toward children. There was no significant main effect of infants’ and toddlers’ communicative behavior toward masked educators on either of the dependent variables. There were significant main effects of country with regard to own language quantity and quality (Table 4). These results were almost compatible when eliminating the item ‘articulation’ from the subscale ‘own language quality’ and the item ‘eye contact’ from the subscale ‘non-verbal communicative behavior in toddlers’ (Supplementary Table S3).

**Table 4:** The linear regression models predicting educators’ own communicative behavior toward children.

	Dependent variables					
	Own language quantity (*n* = 237)		Own language quality (*n* = 237)		Own non-verbal cues (*n* = 237)	
	Estimate (SE)	Value of *p*	Estimate (SE)	Value of *p*	Estimate (SE)	Value of *p*
**Intercept**	**−0.17 (0.05)**	**0.0023**	**1.07 (0.07)**	**<0.001**	**0.68 (0.07)**	**<0.001**
**Verbal communicative behavior** In infants In toddlers	0.02 (0.07) 0.16 (0.09)	0.79 0.065	−0.06 (0.09) − 0.11 (0.11)	0.47 0.31	−0.11 (0.09) −0.04 (0.11)	0.23 0.69
**Non-verbal communicative behavior** In infants In toddlers	0.08 (0.10) −0.04 (0.12)	0.44 0.74	0.08 (0.13) 0.26 (0.15)	0.55 0.079	0.22 (0.13) 0.22 (0.14)	0.077 0.12
**Country (Japanese)**	**0.23 (0.08)**	**0.0033**	**−0.46 (0.10)**	**<0.001**	−0.07 (0.10)	0.44
**Interaction of verbal communicative behavior and country** In infants In toddlers	0.24 (0.16) −0.16 (0.20)	0.15 0.42	−0.14 (0.21) 0.32 (0.25)	0.48 0.21	−0.14 (0.20) 0.12 (0.25)	0.48 0.64
**Interaction of non-verbal communicative behavior and country** In infants In toddlers	−0.00 (0.20) 0.01 (0.29)	0.99 0.96	0.22 (0.26) −0.20 (0.37)	0.38 0.60	0.21 (0.25) −0.03 (0.36)	0.40 0.93
Adjusted *R*^2^	0.10		0.18		0.06	

Values with less than 0.05 of value of *p*s are shown in bold.

**Figure 1:**
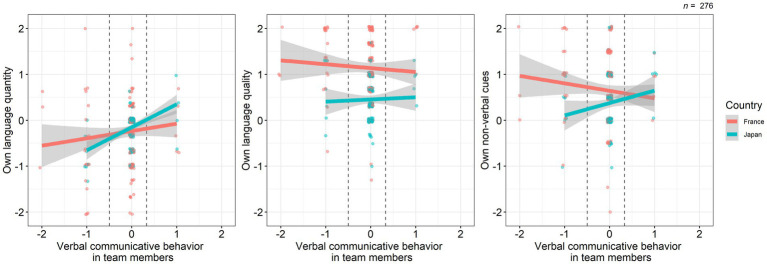
The relationship between educators’ own and team members’ communicative behavior. Dot clusters represent individual data points. The lines and gray zones represent the regression lines and 95% confidence intervals, respectively. Dashed lines represent mean ± 1 SD in verbal communicative behavior in team members.

For the relationship between educators’ own and team members’ behavior, we conducted three linear regression models, with ‘verbal communicative behavior in team members’ as a predictor of each aspect of educators’ own perceived communicative behavior. The main effects of team members’ communicative behavior differed depending on the dependent variables (Figure 1). For the linear regression model predicting own language quantity, we did not find any significant main effects (Table 5). For the model predicting own language quality, we found a significant difference in the country, suggesting that French educators perceived to speak to their colleagues with higher linguistic quality such as better articulation or larger volume than Japanese educators in the context of mask-wearing (Estimate = −0.68, SE = 0.07, *p* < 0.001). For the model predicting own non-verbal cues, we found significant main effects of country (Estimate = −0.27, SE = 0.08, *p* = 0.0011) and the interaction between verbal communicative behavior in team members and country (Estimate = 0.43, SE = 0.22, *p* = 0.049). A *post-hoc* simple slopes analysis for this model demonstrated that, for those who perceived their colleagues came to talk less (−1 SD = −0.50), French educators perceived to enhance non-verbal cues significantly more than Japanese educators (Estimate = −0.48, SE = 0.13, *p* < 0.001). On the other hand, for those who perceived their colleagues came to talk more (+1 SD = 0.33), such perceived enhancement of non-verbal cues did not significantly differ between France and Japan (Estimate = −0.12, SE = 0.12, *p* = 0.29). When focusing on simple slopes for each country, verbal communicative behavior in team members was not significantly related to own non-verbal cues (France: Estimate = −0.16, SE = 0.11, *p* = 0.15; Japan: Estimate = 0.27, SE = 0.19, *p* = 0.15).

**Table 5:** The linear regression models predicting educators’ own communicative behavior toward team members.

	Dependent variables					
	Own language quantity (*n* = 276)		Own language quality (*n* = 276)		Own non-verbal cues (*n* = 276)	
	Estimate (SE)	Value of *p*	Estimate (SE)	Value of *p*	Estimate (SE)	Value of *p*
Intercept	**−0.23 (0.05)**	**<0.001**	**1.14 (0.05)**	**<0.001**	**0.64 (0.06)**	**<0.001**
Verbal communicative behavior in team members	0.16 (0.10)	0.11	−0.08 (0.10)	0.42	−0.16 (0.11)	0.15
Country (Japanese)	0.08 (0.07)	0.26	**−0.68 (0.07)**	**<0.001**	**−0.27 (0.08)**	**0.0011**
Interaction of verbal communicative behavior in team members and country	0.34 (0.19)	0.079	0.13 (0.20)	0.52	**0.43 (0.22)**	**0.049**
Adjusted *R*^2^	0.037		0.24		0.05	

Values with less than 0.05 of value of *p*s are shown in bold.

### Question 2: Relationship between educators’ verbal and non-verbal behavior

To explore whether and how educators’ own perceived verbal communicative behavior affected their non-verbal communicative behavior, we conducted two linear regression models, one addressing communicative behavior toward children and one toward other educators, with ‘own language quality’ and ‘own language quantity’ as predictors of ‘own non-verbal cues.’ For their own communicative behavior toward children, we found a significant positive main effect of own language quality (Estimate = 0.25, SE = 0.07, *p* < 0.001), but the other independent variables including the interactions were not significant (Table 6). This suggests that, regardless of country, the more educators perceived they enhanced their language quality such as articulation or volume when wearing a mask, the more they also perceived using non-verbal cues such as gestures and facial expressions, but they would not increase their amount of language production. The effect of own language quality remained significant even when the item of ‘articulation,’ which had a low value of r.drop, was eliminated from the subscale (Supplementary Table S4).

**Table 6:** The linear regression models predicting educators’ own non-verbal communicative behavior toward others.

	Toward children (*n* = 315)		Toward team members (*n* = 283)	
	Estimate (SE)	Value of *p*	Estimate (SE)	Value of *p*
Intercept	**0.50 (0.09)**	**<0.001**	**0.32 (0.10)**	**0.0022**
Own language quantity	0.06 (0.07)	0.40	0.07 (0.07)	0.35
Own language quality	**0.25 (0.07)**	**<0.001**	**0.31 (0.08)**	**<0.001**
Country (Japanese)	−0.08 (0.12)	0.48	−0.11 (0.13)	0.40
Interaction of own language quantity and country	0.01 (0.16)	0.96	0.02 (0.16)	0.91
Interaction of own language quality and country	0.12 (0.12)	0.31	0.13 (0.14)	0.34
Adjusted *R*^2^	0.08		0.13	

Values with less than 0.05 of value of *p*s are shown in bold.

For participants’ own communicative behavior toward team members, we also found a significant positive main effect of own language quality on own non-verbal cues (Estimate = 0.31, SE = 0.08, *p* < 0.001), but did not find any other significant effects. Thus, toward both children and team members in both countries, participants who perceived they enhanced their language quality also perceived they increased their non-verbal cues when wearing a mask.

### Question 3: Relationship between children’s verbal and non-verbal behavior

Next, we addressed whether and how children’s verbal communicative behavior affected their non-verbal communicative behavior based on participants’ perception. For infants’ behavior, verbal (spoken) communicative behavior significantly predicted non-verbal communicative behavior (Estimate = 0.23, SE = 0.05, *p* < 0.001), but the other fixed effects were not significant (Table 7). This result suggests that, regardless of country, infants who were perceived to enhance their verbal communicative behavior in terms of babbling or repetition were also perceived to increase their non-verbal communicative behavior such as smiling or gestures when educators were wearing a mask.

**Table 7:** The linear regression models predicting children’s non-verbal communicative behavior toward participants.

	Infants (*n* = 246)		Toddlers (*n* = 282)	
	Estimate (SE)	Value of *p*	Estimate (SE)	Value of *p*
Intercept	0.06 (0.05)	0.25	**0.15 (0.03)**	**<0.001**
Verbal communicative behavior	**0.23 (0.05)**	**<0.001**	**0.25 (0.04)**	**<0.001**
Country (Japanese)	−0.00 (0.07)	0.95	**−0.11 (0.05)**	**0.018**
Interaction of verbal communicative behavior and country	0.08 (0.10)	0.43	0.11 (0.09)	0.21
Adjusted *R*^2^	0.11		0.16	

Values with less than 0.05 of value of *p*s are shown in bold.

For communicative behavior in toddlers, we found significant main effects of verbal communicative behavior (Estimate = 0.25, SE = 0.04, *p* < 0.001) and country (Estimate = −0.11, SE = 0.05, *p* = 0.018) on non-verbal communicative behavior, but there was no significant interaction. Thus, similar to the result in infants, enhancement of verbal and non-verbal behavior perceived by educators were positively related. In addition, in toddlers we found a cross-cultural difference such that the extent to which toddlers were perceived to use non-verbal communicative cues was lower in Japan than in France. A supplemental analysis that eliminated the item ‘eye contact’ from the subscale ‘non-verbal communicative behavior’ showed a remaining significant effect of verbal communicative behavior, while the effect of country disappeared (Supplementary Table S5). Considering the supplemental result, the degree of non-verbal communicative behavior in toddlers might be equivalent across countries.

### Question 4: Relationship between educators’ own communicative behavior and their own difficulty of exchange with masked colleagues

In questions 1–3, we explored the relationships of some aspects of communicative behavior in the context of mask-wearing at daycares. We then saw whether and how educators’ feelings of difficulty in exchanging with their colleagues wearing a mask predicted educators’ own communicative behavior toward children (Table 8) and colleagues (Table 9).

**Table 8:** The linear regression models predicting educators’ own communicative behavior toward children.

	Dependent variables					
	Own language quantity (*n* = 281)		Own language quality (*n* = 281)		Own non-verbal cues (*n* = 281)	
	Estimate (SE)	Value of *p*	Estimate (SE)	Value of *p*	Estimate (SE)	Value of *p*
Intercept	−0.02 (0.08)	0.83	**0.59 (0.10)**	**<0.001**	**0.62 (0.11)**	**<0.001**
Own ease of exchange with team members	**0.20 (0.06)**	**0.0023**	**−0.47 (0.08)**	**<0.001**	−0.11 (0.08)	0.17
Country (Japanese)	−0.01 (0.11)	0.95	−0.19 (0.13)	0.14	−0.17 (0.14)	0.20
Interaction of own ease of exchange with team members and country	**−0.25 (0.11)**	**0.019**	0.14 (0.13)	0.27	−0.17 (0.13)	0.21
Adjusted *R*^2^	0.10		0.28		0.03	

Values with less than 0.05 of value of *p*s are shown in bold.

**Table 9:** The linear regression models predicting educators’ own communicative behavior toward team members.

	Dependent variables					
	Own language quantity (*n* = 281)		Own language quality (*n* = 281)		Own non-verbal cues (*n* = 281)	
	Estimate (SE)	Value of *p*	Estimate (SE)	Value of *p*	Estimate (SE)	Value of *p*
Intercept	0.01 (0.11)	0.94	**0.59 (0.10)**	**<0.001**	**0.50 (0.12)**	**<0.001**
Own ease of exchange with team members	**0.22 (0.08)**	**0.0084**	**−0.48 (0.08)**	**<0.001**	−0.12 (0.09)	0.19
Country (Japanese)	−0.06 (0.14)	0.65	**−0.37 (0.13)**	**0.0057**	**−0.37 (0.15)**	**0.016**
Interaction of own ease of exchange with team members and country	−0.07 (0.14)	0.60	0.11 (0.13)	0.40	−0.29 (0.15)	0.059
Adjusted *R*^2^	0.03		0.35		0.07	

Values with less than 0.05 of value of *p*s are shown in bold.

First, we ran three linear regression models, with ‘own ease of exchange with team members’ as a predictor of each aspect of educators’ own communicative behavior toward children. For the model predicting own language quantity toward children, there were significant main effects of own ease of exchange with team members (Estimate = 0.20, SE = 0.06, *p* = 0.0023) and an interaction with country (Estimate = −0.25, SE = 0.11, *p* = 0.019), but no main effect of country. A *post-hoc* simple slopes analysis for this model showed that, for those who felt it more difficult to exchange with masked team members (−1 SD = − 1.3), French educators perceived a significant reduction of the amount of talking to children compared with Japanese educators (Estimate = 0.38, SE = 0.094, *p* < 0.001). On the other hand, for those who felt it less difficult to exchange with masked team members (+1 SD = −0.35), a significant cultural difference was not observed (Estimate = 0.08, SE = 0.080, *p* = 0.31). When focusing on simple slopes for each country, own ease of exchange with masked team members showed a significant positive effect on own language quantity toward children in French educators (Estimate = 0.20, SE = 0.06, *p* = 0.0023), whereas there was no significant relationship between them in Japanese ones (Estimate = −0.05, SE = 0.08, *p* = 0.53). This suggests that only French educators perceived to reduce language quantity toward children when they felt that exchange with masked others was hard. For the model predicting own language quality toward children, we found a significant effect of own ease to exchange with team members (Estimate = −0.47, SE = 0.08, *p* < 0.001). The result remained significant even when ‘articulation’ that had a lower r.drop value was eliminated from the dependent variable (Supplementary Table S6). This suggests that, regardless of country, the more educators felt it difficult to exchange with masked others, the more they perceived to enhance their language quality when wearing a mask. For the model predicting own non-verbal cues toward children, there were not any significant fixed effects.

Next, we performed three linear regression models, with ‘own ease of exchange with team members’ as a predictor of educators’ own communicative behavior toward team members. The results were similar to the models predicting educators’ own communicative behavior toward children. The model predicting own language quantity showed a significant main effect of own ease of exchange with team members (Estimate = 0.22, SE = 0.08, *p* = 0.0084). In contrast with the analysis on language quantity toward children, the interaction with the country was not significant. The model predicting own language quality found significant main effects of own ease of exchange with team members (Estimate = −0.48, SE = 0.08, *p* < 0.001) and country (Estimate = −0.37, SE = 0.13, *p* = 0.0057), suggesting that, similar to the analysis of language quality toward children, educators who felt difficulty in exchanging with colleagues wearing a mask were more likely to perceive that they enhanced their language quality. However, the degree of perceived enhancement was less in Japanese educators than in French ones. Lastly, the model predicting own non-verbal cues showed a significant main effect of the country (Estimate = −0.37, SE = 0.15, *p* = 0.016).

### Question 5: Relationship between educators’ communicative behavior and their perception of others’ difficulty of exchange with masked educators

To address the question of whether and how educators’ feelings about others’ difficulty in masked exchanges affected those educators’ own communicative behavior, we ran six linear regression models, three addressing the relationship between children’s perceived difficulty and educators’ behavior toward children and the other three addressing that between team members’ perceived difficulty and educators’ behavior toward team members.

**Figure 2:**
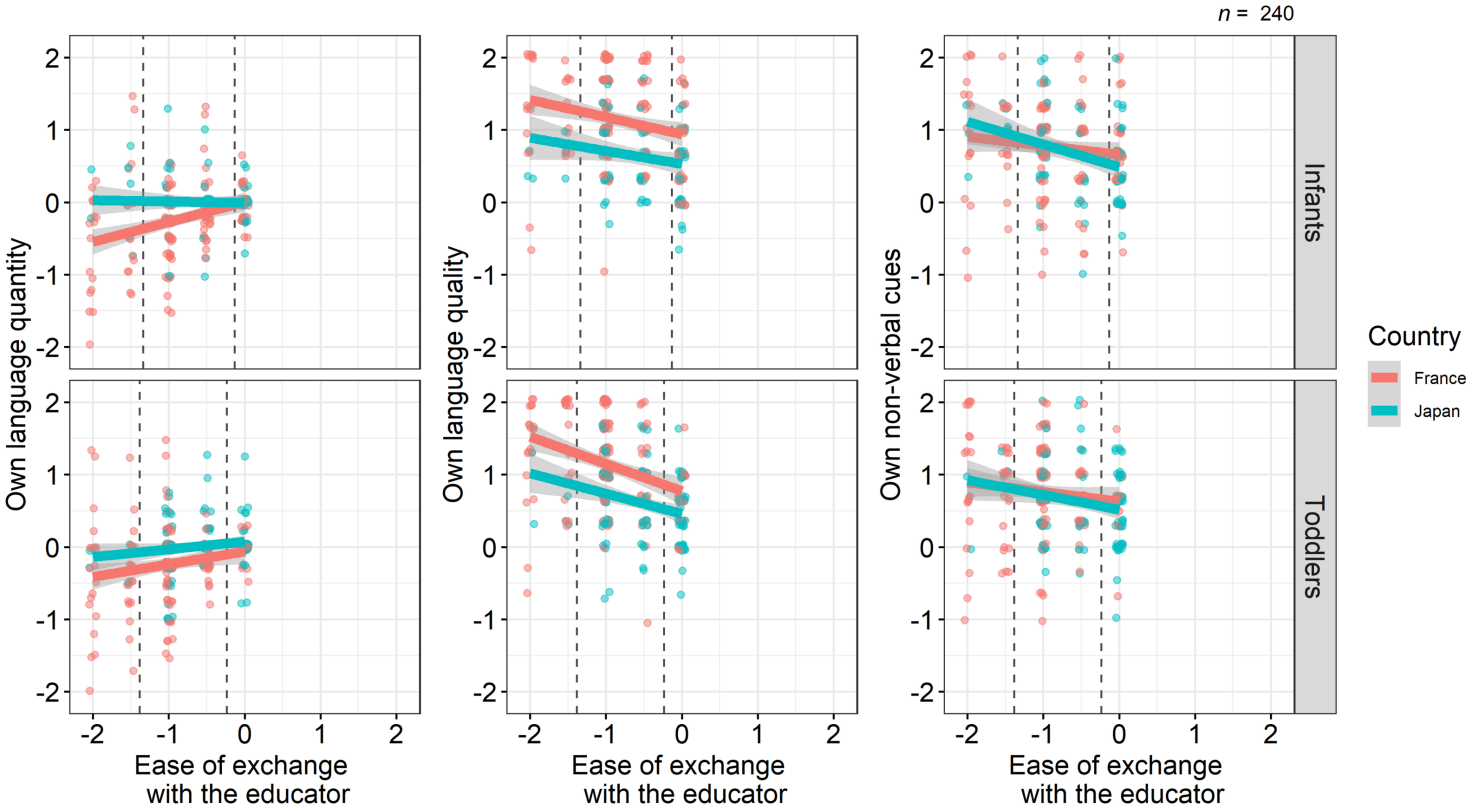
The relationship between children’s ease of exchange and educators’ own communicative behavior. Dot clusters represent individual data points. The lines and gray zones represent the regression lines and 95% confidence intervals, respectively. Dashed lines represent mean ± 1 SD in ease of exchange with the educator.

For the model predicting own language quantity toward children, there were a significant main effect of ease of exchange with the educator for infants (Estimate = 0.27, SE = 0.07, *p* < 0.001) and an interaction with country (Estimate = −0.36, SE = 0.13, *p* = 0.0077), but there were not main effects of country and ease of exchange with the educator for toddlers (Figure 2; Table 10). A *post-hoc* simple slopes analysis showed that, when infants were thought to have more difficulty in exchanging with masked educators (−1 SD = −1.3), French educators felt they significantly reduced their language quantity compared with Japanese educators (Estimate = 0.52, SE = 0.19, *p* = 0.0053). On the other hand, when infants were thought to have less difficulty (+1 SD = −0.13), there was no significant cultural difference in reduction of language quality (Estimate = 0.08, SE = 0.11, *p* = 0.43). When focusing on simple slopes for each country, ease of exchange with masked educators showed a significant positive effect on own language quantity toward children in French educators (Estimate = 0.27, SE = 0.07, *p* < 0.001), whereas there was no significant relationship between them in Japanese ones (Estimate = −0.10, SE = 0.11, *p* = 0.39). This suggests that only French educators perceived to reduce language quantity toward infants when they felt that infants had a difficulty of exchange with masked educators. For the model predicting own language quality toward children, we found significant main effects of ease to exchange with the educator for toddlers (Estimate = −0.31, SE = 0.10, *p* = 0.0025) and country (Estimate = −0.29, SE = 0.13, *p* = 0.031), but not for infants. However, removing ‘articulation’ that had a lower r.drop value from the dependent variables, ease of exchange with the educator for both infants and toddlers became significant, and the cultural difference became not significant (Supplementary Table S7). Thus, what is at least consistent for analysis between with and without this item is that the more children seemed to experience difficulty in exchanging with the masked educators, the more educators perceived to make their language quality salient. For the model predicting ‘own non-verbal cues toward children’, there were not any significant fixed effects.

**Table 10:** The linear regression models predicting educators’ own communicative behavior toward children.

	Dependent variables					
	Own language quantity (*n* = 240)		Own language quality (*n* = 240)		Own non-verbal cues (*n* = 240)	
	Estimate (SE)	Value of *p*	Estimate (SE)	Value of *p*	Estimate (SE)	Value of *p*
**Intercept**	−0.01 (0.08)	0.88	**0.76 (0.10)**	**<0.001**	**0.60 (0.10)**	**<0.001**
**Ease of exchange with the educator** For infants For toddlers	**0.27 (0.07)** −0.01 (0.08)	**<0.001** 0.94	−0.08 (0.09) −**0.31 (0.10)**	0.41 **0.0025**	−0.10 (0.10) −0.07 (0.11)	0.28 0.50
**Country (Japanese)**	0.04 (0.11)	0.74	**−0.29 (0.13)**	**0.031**	−0.15 (0.14)	0.29
**Interaction of ease of exchange with the educator and country** For infants For toddlers	**−0.36 (0.13)** 0.15 (0.15)	**0.0077** 0.34	0.06 (0.17) 0.01 (0.19)	0.72 0.94	−0.16 (0.17) −0.04 (0.20)	0.37 0.83
Adjusted *R*^2^	0.11		0.21		0.04	

Values with less than 0.05 of value of *p*s are shown in bold.

The models with ‘team members’ ease of exchange with the educator’ as a predictor of educators’ own communicative behavior toward team members showed similar results to those regarding communication with children (Table 11). For the model predicting own language quantity, there was a significant effect of ease of exchange with the educator (Estimate = 0.23, SE = 0.08, *p* = 0.0040). For the model predicting own language quality, we found significant effects of ease of exchange with the educator (Estimate = −0.52, SE = 0.07, *p* < 0.001) and the interaction with the country (Estimate = 0.25, SE = 0.12, *p* = 0.036). According to a *post-hoc* simple slopes analysis, when team members were thought to have more difficulty exchanging with masked participants (−1 SD = −1.60), French educators felt they enhanced their language quality significantly more than Japanese educators (Estimate = −0.60, SE = 0.13, *p* < 0.001), and even when team members were felt to have less difficulty (+1 SD = −0.26), the cultural difference remained significant although the difference was smaller (Estimate = −0.26, SE = 0.10, *p* = 0.012). When focusing on simple slopes for each country, ease of exchange for team members showed a significant negative effect on own language quality in both French (Estimate = −0.52, SE = 0.07, *p* < 0.001) and Japanese educators (Estimate = −0.27, SE = 0.10, *p* = 0.0058). For the model predicting own non-verbal cues, there was a significant main effect of ease of exchange with the educator (Estimate = −0.21, SE = 0.08, *p* = 0.012). Overall, in terms of perceived difficulty of exchange for team members, the more team members seemed to have difficulty exchanging with the educators, the more educators felt they enhanced both language quality and non-verbal cues while, at the same time, reducing language quantity.

**Table 11:** The linear regression models predicting educators’ own communicative behavior toward team members.

	Dependent variables					
	Own language quantity (*n* = 281)		Own language quality (*n* = 281)		Own non-verbal cues (*n* = 281)	
	Estimate (SE)	Value of *p*	Estimate (SE)	Value of *p*	Estimate (SE)	Value of *p*
Intercept	0.02 (0.11)	0.82	**0.50 (0.10)**	**<0.001**	**0.39 (0.12)**	**0.0010**
Ease of exchange with the educator for team members	**0.23 (0.08)**	**0.0040**	**−0.52 (0.07)**	**<0.001**	**−0.21 (0.08)**	**0.012**
Country (Japanese)	−0.14 (0.13)	0.29	−0.19 (0.12)	0.12	−0.23 (0.14)	0.10
Interaction of ease of exchange with the educator for team members and country	−0.14 (0.13)	0.27	**0.25 (0.12)**	**0.036**	−0.19 (0.14)	0.16
Adjusted *R*^2^	0.03		0.38		0.10	

Values with less than 0.05 of value of *p*s are shown in bold.

### Question 6: Relationship between educators’ attitude toward wearing a mask and their perceived difficulty of exchange

We addressed whether and how educators’ attitudes toward wearing a mask (i.e., positive or negative feelings) related to their perceived difficulty of exchange (Table 12). We constructed four linear regression models with educators’ ‘own attitude toward wearing a mask’ as a predictor of each difficulty subscale (i.e., ‘own ease of exchange with team members,’ ‘team members’ ease of exchange with the educator,’ ‘infants’ ease of exchange with the educator,’ and ‘toddlers’ ease of exchange with the educator’). The models showed that the more educators had a negative attitude toward wearing a mask, the more likely they were to perceive conversational exchange as difficult, regardless of dependent variables. Additionally, the model revealed that, for team members’ and toddlers’ ease of exchange with the educators, there was a significant effect of country, suggesting that French educators were more likely to think that others should have had difficulty in exchanging with them in the context of mask-wearing compared with Japanese educators, but such cultural difference was not observed when dependent variable was ‘own ease of exchange with team members.’

**Table 12:** The linear regression models predicting ease of exchange.

	Dependent variables							
	Own ease of exchange with team members (*n* = 263)		Ease of exchange with the educator for team members (*n* = 261)		Ease of exchange with the educator for infants (*n* = 222)		Ease of exchange with the educator for toddlers (*n* = 260)	
	Estimate (SE)	*p*-value	Estimate (SE)	*p*-value	Estimate (SE)	*p*-value	Estimate (SE)	*p*-value
Intercept	**−1.46 (0.08)**	**<0.001**	**−1.54 (0.09)**	**<0.001**	**−1.26 (0.09)**	**<0.001**	**−1.36 (0.08)**	**<0.001**
Own attitude toward wearing a mask	**0.15 (0.04)**	**<0.001**	**0.14 (0.04)**	**<0.001**	**0.21 (0.04)**	**<0.001**	**0.19 (0.04)**	**<0.001**
Country (Japanese)	0.32 (0.18)	0.082	**0.71 (0.20)**	**< 0.001**	0.25 (0.24)	0.30	**0.57 (0.18)**	**0.0021**
Interaction of own attitude toward wearing a mask and country	0.04 (0.07)	0.56	−0.03 (0.08)	0.71	−0.05 (0.09)	0.59	−0.09 (0.07)	0.22
Adjusted *R*^2^	0.27		0.33		0.15		0.24	

Values with less than 0.05 of value of *p*s are shown in bold.

### Question 7: Relationship between educators’ own knowledge about language development and their worry about children’s development

We hypothesized that a better understanding of language development might have mitigated worry about the impact of daily mask-wearing on children’s development. However, the linear regression model predicting the worry measure did not find any significant effects of ‘own knowledge about language development’ (Table 13).

**Table 13:** The linear regression model predicting educators’ own worry about children’s development.

	Estimate (SE)	*p*-value
Intercept	18.34 (15.75)	0.25
Own knowledge about language development	1.52 (0.94)	0.11
Country (Japanese)	14.94 (20.26)	0.46
Interaction between knowledge about language development and country	−0.68 (1.27)	0.60
Adjusted *R*^2^	0.003	

Values with less than 0.05 of value of *p*s are shown in bold. *n* = 260.

## Discussion

Daily mask wearing might affect children’s speech input by changes in interlocutors’ communicative behavior. In order to explore how such changes might manifest, we surveyed Early Childhood Educators, a population that regularly interacts with children through a face mask since the onset of the pandemic, on perceived changes in communicative behaviors while wearing face masks in daycare settings. We surveyed daycare educators from two different cultural contexts, Japan and France.

### Perceived changes in daily communicative behaviors

Our first goal was to determine whether and what kind of changes educators perceived in the different aspects of their daily communicative interactions when masks were worn. We found that educators did perceive such changes for many aspects of their communicative behavior.

Regarding our surveyed educators’ own communicative behavior, they perceived a decrease in language quantity toward others in both countries (toward children and their team for French educators, and only toward team members for Japanese educators). In contrast, an increase in language quality and non-verbal cues were reported across the board.

Although these are only reported behaviors, this could indicate that the total amount of language input children receive – both direct and overheard input – could have decreased in the context of mask-wearing, potentially affecting language development (Akhtar, 2005; Shneidman et al., 2009; Gampe et al., 2012; but see Weisleder and Fernald, 2013). On the other hand, the reported increases in language quality and non-verbal cues could compensate for this reduction of language quantity^5^, and reflect educators’ desire to communicate with others more smoothly, as seen with the use of Lombard or clear speech (Krause and Braida, 2004; Brumm and Zollinger, 2011).

Educators reported a decrease in children’s verbal communication in both countries. This result suggests that not only adults but also even young children might have changed their communicative behavior, although it is unclear whether they would have just been influenced by educators’ changing ways of interacting with them or if they would have actively modulated their communicative styles. Previous literature indicates that young children are already capable of modulating how they interact in accordance with their interlocutor’s style of communication (Shatz and Gelman, 1973; Syrett and Kawahara, 2014). No change was reported regarding children’s non-verbal behavior. The absence of a negative effect on non-verbal behavior might indicate that it is less likely impacted by masked communication, or that it is more easily compensated for. On the other hand, the absence of a positive increase of children’s non-verbal behavior like the one seen in educators, might indicate that their communicative modulation abilities are not yet functional enough to enhance their non-verbal communicative behavior to compensate for their reduced verbal communicative behavior.

### Relationship between educators’ and their interlocutors’ behaviors (Question 1)

Educators’ own perceived communicative behavior was not significantly related to their perception of children’s communicative behavior; however, our results suggest a link to their team members’ communicative behavior.

This might indicate that educators in both countries, as professionals, attempted to ensure and keep the quantity and quality of their interactions with children as equal as possible (or perceived they were doing so), regardless of children’s behavior. It is also possible that these results reflect a leader-follower dynamic in the relationship between educators and children, by which the educator sets the tone and is thus less influenced by changes in children’s behavior. On the other hand, the results regarding communication with team members might be reflecting educators’ natural tendency to adapt their interaction style when wearing a mask.

### Relationship between subtypes of communicative behaviors (Question 2, 3)

Educators’ reported communicative behaviors toward children and their team were consistent: regardless of the interlocutor, an increase in language quality was linked to an increase in non-verbal cues, but not to language quantity. Such a pattern might indicate that language quality and non-verbal cues are both perceived as cues that could serve a compensatory role during masked communication.

Regarding children’s reported behavior, be it infants or toddlers, our results show a positive link between educators’ perception of their verbal and non-verbal behavior. This could imply, when added to the lack of an increase of children’s non-verbal behavior (see above), that children whose verbal communicative behavior was weak also produced weakened non-verbal cues, which might lead to the reduction of opportunities to interact with others.

### Attitudes toward mask, their added difficulty and impact on communicative behaviors (Question 4, 5, 6)

We also examined other factors apart from educators’ and others’ communicative behaviors, which might have influenced educators’ communication. Firstly, educators perceived a decrease in the ease of exchange overall (in children, team members and themselves), compared to a no-mask situation. Our results demonstrated a link between educator’s attitude toward mask wearing (regarding usefulness, comfort), and how easy they perceived masked communication to be for themselves and for others: the less educators had a positive attitude toward masks and the more they reported a decrease in ease of exchange.

Secondly, this perceived ease of exchange appeared to be linked to communicative behaviors: a decrease in ease of exchange (in the educators or in others), was linked to a decrease in the quantity and an increase in the quality of the language the educators addressed to their interlocutors. It might be the case that educators thought that it was somewhat less worthwhile to have a conversation with others because it was hard to understand them or to make themselves understood by others. On the other hand, the increase in language quality might demonstrate the educators’ willingness to adapt to their interlocutors’ potential difficulty in the exchange (Hazan and Baker, 2011; Tellier et al., 2021). This could explain the fact that the less educators perceived children at ease with masked communication, the more they increased their language quality.

### Country differences

The reported changes in educators’ and others communicative behaviors had overall a similar direction for both countries. The main difference was in the strength of those changes, which were stronger in the French sample. Similarly, the level of reported ease of exchange in educators and others in masked communication was consistently lower in the French sample, and French educators had significantly more negative attitudes toward mask wearing. The strength of these feelings may be related to familiarity with daily mask-wearing, but the underlying perceived difficulty may be difficult to overcome, as even Japanese educators experienced difficulties exchanging with others.

On the other hand, it may also be that the observed differences between the two countries do not solely derive from a differential impact of masks on communication but from differences predating the introduction of facemasks in daycares’ daily lives. Although it was not conducted in daycares and it compared Japanese to American mothers, the work of Fogel et al. (1988) and Toda et al. (1990), has indeed identified systematic differences in how they interacted with their infants. For example, the former shows a more frequent use of movement and touch, as well as onomatopoeia, while the latter shows a higher reliance on facial expressions, accompanied by an increased use of questions. Those differences in adults’ communicative behavior were also accompanied by differences in infants’ communicative behavior (e.g., longer duration for smiling and vocalizing).

In addition to differences in communicative behaviors in children and adults, differences have been noted in the way socio-linguistic signals might be processed. Indeed, Easterners and Westerners seem to show different patterns of face-scanning behaviors, with Easterners spending more time on the central and mouth region of the face, and Westerners looking generally more toward the eye region, be it during simple face observation (Senju et al., 2013), facial emotion recognition (Caldara, 2017), or social interactions (Haensel et al., 2020).

These observed differences among cultures, predating the use of facemasks, might thus play a role in the cultural differences in our results, by either having contributed to a baseline difference in communicative behaviors prior to the pandemics, or by modulating the way locutors were impacted by masked communication and how easily they could adapt and generate compensatory behaviors.

### Limitations and future directions

The data we obtained consisted of educators’ perceptions and not of direct observations of communication at daycares. Although a direct assessment of educators’ and children’s communicative behavior would be ideal, such a dataset is near-impossible to obtain: Mask mandates in daycares were implemented very soon after the begin of the pandemic, and we cannot go back in time to assess communicative behavior before this mandate. The present data and results thus represent an important step toward a better understanding of changes that could occur in communication around young children when masks are worn. A possible future direction would be to investigate the discrepancy between perceptual and actual communicative behavioral levels, and whether educators’ feelings and attitudes mediate the gap between these two levels.

It should also be taken into account that the systematization of mask wearing in daycares was accompanied by other environmental changes (e.g., a higher general level of stress in educators, social distancing among adults, and increased rotation in teams’ compositions). These factors might have contributed to the communicative changes reported here.

Some of our subscales had low internal consistency, which might affect the validity of the summarized variables. However, results after elimination of problematic items were by and large consistent with those of the original items, supporting the robustness of our findings, and at the very least transparently demonstrating where our survey might be lacking. The purpose of the present study was to explore changes in communication in the context of mask-wearing. We encourage further validation and improvement on this first run of the questionnaire, which is freely available on our OSF page.^6^

Our sample came predominantly from urban areas, and would benefit from comparisons with non-urban areas, which might have been differently impacted with COVID-19. Similarly, although we tried to match our daycare populations for our two countries of study, French daycares were overall smaller than Japanese ones, which might explain why the proportion of directors surveyed was significantly larger in France. Although this might have had an impact, as directors have the same background and also work in contact with the children, they should hold similar views to educators regarding masks impacts.^7^

With regard to future work, an interesting avenue should be to look at children at risk of language development delays. For example, mask impacts could manifest differently for children with hearing loss, who are highly dependent on articulatory cues (Charney et al., 2021). These differences could be in the way children react to the presence of a mask on educators’ faces, or also in how educators specifically modify their communicative behavior when interacting with them, maybe demonstrating some kind of specific compensatory mechanism. A recent study indeed indicates that educators might adapt their speech style differently depending on children’s developmental risk, such that measures of speech quantity and quality to children with hearing impairment showed a tendency to increase more than to children without hearing impairment (Mitsven et al., 2022).

The present findings imply several possible factors that should be taken into account when thinking about children’s language development behind masks. First, the total amount of language input children receive could decrease in the context of mask-wearing. This includes not only input directly aimed toward children but also overheard input. As the quantity of language input relates to children’s language development (Hoff, 2003, 2006; Weisleder and Fernald, 2013), educators might be encouraged to think about whether conversations around children become too rare. In particular, perceived language quantity toward children was lower in France than in Japan. Theoretically, such a cultural comparison is interesting to investigate how changes in input quantity affect language development. Second, educators might use compensatory strategies when wearing a mask, that is, they could enhance language quality and/or non-verbal cues. Based on the results from this study, educators in both countries reported enhancing these factors, and language quality such as volume or articulation was especially thought to be adjusted according to the difficulty of exchange for either educators themselves or listeners. By deliberately leveraging such factors, educators could make interactions with infants/toddlers more valuable and even scaffold children’s language development. Meanwhile, even if such compensatory strategies go beyond just perceptions, the question is still open as to what kind of specific quality adjustment takes place in masked communication, and whether the enhancement educators naturally convey is sufficient enough for children to learn languages.

While we focused on the impact of mask wearing on speech, emotion perception is also important for communication and might be impacted by mask wearing. Indeed, emotion perception is influenced by the visibility of certain parts of the face, with adults relying mostly on the eye and mouth regions when inferring an emotion. Interestingly, the importance of each face region varies depending on the emotion to be inferred, and while adults attend more to the eye region to recognize sadness or fear, they rely more on the mouth region to recognize disgust or happiness (Calder et al., 2000; Schurgin et al., 2014; Wegrzyn et al., 2017). Thus, mask wearing, in hiding only the lower part of the face, might impact emotion perception differentially depending on the emotion to be inferred.

Similarly with language processing, it has been shown that different regions of the face might play different roles. For instance, while the facial articulatory cues in the lower part of the face may be more important in lexical decision tasks, the ones taking place in the upper part of the face, such as eyebrow raising or eye-widening, might play a bigger role in prosody perception (Swerts and Krahmer, 2010). Future studies should focus more on the potential impact of emotions production and perception on communication in context of mask wearing, for instance using frameworks for measuring the facial articulatory cues used in emotions such as EMFACS (Friesen and Ekman, 1983).

### Conclusion

We did not examine the actual effects on the language development of the children, nor did we examine the actual behavior of the caregivers. To our knowledge, however, this is the first cross-cultural study to document what kind of changes caregivers perceive to have occurred in their daily behavior at daycares, and we believe that this is a good beginning for future research focusing on language development behind masks. The results drawn in this study suggest the possibility that children’s language-learning experiences may have been affected by mask-wearing in many different aspects. If these findings were beyond just educators’ perception, wearing a mask not only leads to reduction of visual–auditory sensory input but also modifications of language quantity, language quality, and non-verbal cues children receive, and such changes would be also dependent on educators’ feelings and attitudes toward masks. A broad perspective concerning communication in the context of mask-wearing will enrich language development theories and childcare practices.

## Data availability statement

The datasets presented in this study can be found in online repositories. The names of the repository/repositories and accession number(s) can be found at: https://osf.io/xtjeb.

## Ethics statement

The studies involving human participants were reviewed and approved by Office for Life Science Research Ethics and Safety, The University of Tokyo. The patients/participants provided their written informed consent to participate in this study.

## Author contributions

CC, MB, and ST designed the research. HH translated the questionnaire in Japanese. CC and EA translated the questionnaire in French and implemented the questionnaire online. HH performed the analyses. CC and HH wrote the first draft of the manuscript. ST supervised the study. All authors created and validated the questionnaire, contributed to the article, and approved the submitted version.

## Funding

This research was supported by a Fyssen Foundation grant and Japan Society for the Promotion of Science post-doctoral fellowship grant P20722 to MB, JSPS KAKENHI Grant Number JP21J00750 to HH, a Jacobs Foundation Fellowship to ST, a research grant by the Institute for AI and Beyond to ST, JSPS KAKENHI Grant Number 20H05617 to ST, JSPS KAKENHI Grant Number 20H05919 to ST, the World Premier International Research Center Initiative (WPI), MEXT, Japan, the Center for Early Childhood Development, Education, and Policy Research (Cedep), the University of Tokyo, and by Agence Nationale pour la Recherche (ANR-17-EURE-0017, ANR-10-IDEX-0001-02). CC acknowledges the “École Doctorale Frontières de l’Innovation en Recherche et Éducation – Programme Bettencourt” for financial support.

## Conflict of interest

HH and ST are affiliated with a research institute at the University of Tokyo which is funded by Softbank Group. The authors receive research funding in this institute’s basic research tier through this affiliation, which has the non-commercial aim of developing new research fields. NA is a co-founder and partner at Rising Up.

The remaining authors declare that the research was conducted in the absence of any commercial or financial relationships that could be construed as a potential conflict of interest.

## Publisher’s note

All claims expressed in this article are solely those of the authors and do not necessarily represent those of their affiliated organizations, or those of the publisher, the editors and the reviewers. Any product that may be evaluated in this article, or claim that may be made by its manufacturer, is not guaranteed or endorsed by the publisher.

## Footnotes

^1^ https://www.qualtrics.com

^2^ https://osf.io/xtjeb/

^3^We also confirmed if there were some extreme participants who chose the same value for all the items from Subscales I to VII. Only two participants from the Japanese sample chose 0 (i.e., the ‘no-change’ option) for all of them; however, the analyses after omitting these two did not change any of the results reported here in terms of statistical significance.

^4^For the diagnostic visualization of linear regressions can be seen on our OSF page https://osf.io/xtjeb/.

^5^However, the correlations between language quantity and language quality or paralinguistic cues were quite small, ranging from - 0.08 to 0.15 (see Supplementary Table S8 for details).

^6^ https://osf.io/xtjeb/

^7^Although it would be interesting to see whether educators’ perceptions differed depending on the job position, the comparison of their perceptions between directors and non-directors may lead to sampling bias because the sample size for each job position considerably varied. For reference, we calculated the means and standard deviations for each subscale score for directors and non-directors. We observed two significant differences in the French sample, ‘own ease of exchange with team members’ (Non-directors perceived more difficulty in interacting with their colleagues compared with directors) and ‘ease of exchange with the educator for toddlers’ (Non-directors were more likely to perceive that toddlers seemed to have difficulty in interacting with them compared with directors). See Supplementary **Table S9** for more details.
