# Supplementary material for: Mask wearing in Japanese and French nursery schools: The perceived impact of masks on communication
Source: Front Psychol. 2022 Nov 7;13:874264. doi: 10.3389/fpsyg.2022.874264 (PMC9677818; doi:10.3389/fpsyg.2022.874264)
Supplement: Supplementary file 1 [file Data_Sheet_1.pdf]

Supplementary Material

Table S1

Comparison of Subscale Scores Against the 'No-Change' Option

Subscale	French				Japanese			
	Mean (SD)	*t*	Adj.*p*	*d*	Mean (SD)	*t*	Adj.*p*	*d*
Own language quantity								
Toward children	**-0.25 (0.58)**	-5.77	< 0.001	0.43	0.01 (0.35)	0.43	1.0	0.04
Toward team members	**-0.26 (0.73)**	-4.46	< 0.001	0.36	**-0.15 (0.40)**	-4.34	< 0.001	0.38
Own language quality								
Toward children	**1.15 (0.63)**	24.09	< 0.001	1.81	**0.62 (0.51)**	14.32	< 0.001	1.22
Toward team members	**1.15 (0.68)**	21.16	< 0.001	1.69	**0.45 (0.50)**	10.16	< 0.001	0.90
Own paralinguistic cues								
Toward children	**0.77 (0.61)**	16.57	< 0.001	1.25	**0.64 (0.54)**	14.01	< 0.001	1.19
Toward team members	**0.65 (0.73)**	11.14	< 0.001	0.89	**0.39 (0.57)**	7.75	< 0.001	0.69
Own ease of exchange with team members	**-1.17 (0.57)**	-25.70	< 0.001	2.06	**-0.65 (0.48)**	-15.23	< 0.001	1.36
Verbal communicative behavior								
In infants	**-0.63 (0.69)**	-11.40	< 0.001	0.92	**-0.23 (0.52)**	-4.20	< 0.001	0.44
In toddlers	**-0.46 (0.60)**	-9.60	< 0.001	0.77	**-0.14 (0.40)**	-3.96	0.0010	0.35
In team members	**-0.14 (0.48)**	-3.58	0.0032	0.29	-0.02 (0.31)	-0.58	1.0	0.05
Non-verbal communicative behavior								
In infants	-0.09 (0.52)	-2.26	0.15	0.18	-0.04 (0.38)	-0.92	1.0	0.09
In toddlers	0.04 (0.44)	1.17	1.0	0.09	-0.01 (0.25)	-0.51	1.0	0.05
Ease of exchange with the educator								
For infants	**-0.85 (0.61)**	-17.25	< 0.001	1.39	**-0.56 (0.55)**	-9.68	< 0.001	1.02
For toddlers	**-1.00 (0.55)**	-22.88	< 0.001	1.81	**-0.54 (0.50)**	-12.14	< 0.001	1.07
For team members	**-1.25 (0.61)**	-25.76	< 0.001	2.06	**-0.53 (0.52)**	-11.42	< 0.001	1.02

Notes. Scores are shown in bold when they significantly differed from the 'no-change' option (i.e., 0). Holm's method was used for the *p*-value adjustment. Effect sizes were calculated using Cohen's *d*.

Table S2
Comparison of Subscale Scores between the Countries

Subscale	French	Japanese	Statistics			Comparison
	Mean (SD)	Mean (SD)	*t*	Adj. *p*	*d*	
Own language quantity						
Toward children	-0.25 (0.58)	0.01 (0.35)	-5.01	< 0.001	0.54	Fr < Ja
Toward team members	-0.26 (0.73)	-0.15 (0.40)	-1.59	0.45	0.18	n.s.
Own language quality						
Toward children	1.15 (0.63)	0.62 (0.51)	8.20	< 0.001	0.911	Fr > Ja
Toward team members	1.15 (0.68)	0.45 (0.50)	9.94	< 0.001	1.15	Fr > Ja
Own paralinguistic cues						
Toward children	0.77 (0.61)	0.64 (0.54)	1.90	0.29	0.21	n.s.
Toward team members	0.65 (0.73)	0.39 (0.57)	3.35	0.0064	0.39	Fr > Ja
Own ease of exchange with team members	-1.17 (0.57)	-0.65 (0.48)	-8.28	< 0.001	0.98	Fr < Ja
Verbal communicative behavior						
In infants	-0.63 (0.69)	-0.23 (0.52)	-5.18	< 0.001	0.64	Fr < Ja
In toddlers	-0.46 (0.60)	-0.14 (0.40)	-5.34	< 0.001	0.61	Fr < Ja
In team members	-0.14 (0.48)	-0.02 (0.31)	-2.56	0.066	0.30	n.s.
Non-verbal communicative behavior						
In infants	-0.09 (0.52)	-0.04 (0.38)	-1.01	0.63	0.12	n.s.
In toddlers	0.04 (0.44)	-0.01 (0.25)	1.26	0.62	0.14	n.s.
Ease of exchange with the educator						
For infants	-0.85 (0.61)	-0.56 (0.55)	-3.74	0.0019	0.48	Fr < Ja
For toddlers	-1.00 (0.55)	-0.54 (0.50)	-7.49	< 0.001	0.88	Fr < Ja
For team members	-1.25 (0.61)	-0.53 (0.52)	-10.80	< 0.001	1.27	Fr < Ja
Own worry about children's development	43.7 (29.2)	45.6 (32.6)	-0.50	0.63	0.06	n.s.
Own language development knowledge	16.61 (2.73)	14.52 (3.32)	5.49	< 0.001	0.69	Fr > Ja
Own attitude towards mask wearing	1.93 (1.11)	2.62 (0.77)	-5.91	< 0.001	0.71	Fr < Ja

Notes. Holm's method was used for the *p*-value adjustment. Effect sizes were calculated using Cohen's *d*.

Table S3

The Linear Regression Models Predicting Children's Non-Verbal Communicative Behavior toward Participants

	Toddlers (n = 282)	
	Estimate (SE)	p
Intercept	0.06 (0.03)	0.09
Verbal communicative behavior	**0.40 (0.05)**	**< 0.001**
Country (Japanese)	-0.04 (0.05)	0.43
Interaction of verbal communicative behavior and country	-0.03 (0.09)	0.70
Adjusted R^2	0.27	

Notes. Values with less than 0.05 of p-values are shown in bold. The item of 'eye contact' (r.drop = 0.05) was eliminated from the subscale 'Non-verbal communicative behavior,' which led to an increase in the internal consistency (alpha changed from 0.35 to 0.42).

Table S4

The Linear Regression Models Predicting Educators' Own Communicative Behavior toward Children

	Dependent variables					
	Own language quantity (n = 237)		Own language quality* (n = 237)		Own paralinguistic cues (n = 237)	
	Estimate (SE)	p	Estimate (SE)	p	Estimate (SE)	p
Intercept	**-0.17 (0.05)**	**0.0016**	**1.16 (0.07)**	**< 0.001**	**0.69 (0.07)**	**< 0.001**
Verbal communicative behavior						
in infants	0.02 (0.07)	0.79	-0.09 (0.09)	0.31	-0.11 (0.09)	0.19
in toddlers	**0.18 (0.09)**	**0.040**	-0.18 (0.12)	0.13	-0.07 (0.11)	0.54
Non-verbal communicative behavior						
In infants	0.12 (0.10)	0.23	0.09 (0.13)	0.49	**0.24 (0.12)**	**0.049**
In toddlers**	-0.12 (0.11)	0.28	0.02 (0.15)	0.88	0.21 (0.14)	0.13
Country (Japanese)	**0.24 (0.08)**	**0.0021**	**-0.47 (0.10)**	**< 0.001**	-0.07 (0.09)	0.45
Interaction of verbal communicative behavior and country						
In infants	0.26 (0.17)	0.11	-0.19 (0.22)	0.38	-0.06 (0.21)	0.78
In toddlers	-0.17 (0.20)	0.39	0.43 (0.26)	0.098	0.22 (0.24)	0.37
Interaction of non-verbal communicative behavior and country						
In infants	0.04 (0.21)	0.84	0.08 (0.28)	0.77	0.45 (0.27)	0.097
In toddlers**	-0.07 (0.30)	0.81	0.01 (0.39)	0.97	-0.60 (0.37)	0.10
Adjusted R^2	0.11		0.18		0.07	

Notes. Values with less than 0.05 of p-values are shown in bold. *The item of 'articulation' (r.drop = 0.30) was eliminated, which led to an increase in the internal consistency (alpha changed from 0.56 to 0.60). **The item of 'eye contact' (r.drop = 0.05) was eliminated, which led to an increase in the internal consistency (alpha changed from 0.35 to 0.42).

Table S5
The Linear Regression Models Predicting Educators' Own Non-Verbal Communicative Behavior toward Others

	Towards children (*n* = 315)	
	Estimate (SE)	*p*
Intercept	**0.54 (0.10)**	**< 0.001**
Own language quantity	0.06 (0.07)	0.38
Own language quality*	**0.19 (0.06)**	**0.0038**
Country (Japanese)	-0.06 (0.12)	0.66
Interaction of own language quantity and country	0.05 (0.16)	0.78
Interaction of own language quality* and country	0.04 (0.11)	0.75
Adjusted R^2	0.05	

Notes. Values with less than 0.05 of *p*-values are shown in bold. *The item of 'articulation' (*r*.drop = 0.30) was eliminated, which led to an increase in the internal consistency (alpha changed from 0.56 to 0.60).

Table S6
The Linear Regression Models Predicting Educators' Own Communicative Behavior toward Children

	Dependent variables	
	Own language quality* (n = 281)	
	Estimate (SE)	p
Intercept	**0.55 (0.10)**	**< 0.001**
Own ease of exchange with team members	**-0.61 (0.08)**	**< 0.001**
Country (Japanese)	-0.13 (0.13)	0.32
Interaction of own ease of exchange with team members and country	0.22 (0.12)	0.081
Adjusted R^2	0.37	

Notes. Values with less than 0.05 of p-values are shown in bold. *The item of 'articulation' (r.drop = 0.30) was eliminated, which led to an increase in the internal consistency (alpha changed from 0.56 to 0.60).

Table S7
The Linear Regression Models Predicting Educators' Own Communicative Behavior toward Children

	Dependent variables	
	Own language quality* (n = 240)	
	Estimate (SE)	p
Intercept	**0.73 (0.09)**	**< 0.001**
Ease of exchange with the educator		
For infants	**-0.22 (0.09)**	**0.013**
For toddlers	**-0.36 (0.10)**	**< 0.001**
Country (Japanese)	-0.25 (0.13)	0.054
Interaction of ease of exchange with the educator and country		
For infants	0.11 (0.16)	0.50
For toddlers	-0.01 (0.18)	0.95
Adjusted R^2	0.31	

Notes. Values with less than 0.05 of p-values are shown in bold. *The item of 'articulation' (r.drop = 0.30) was eliminated, which led to an increase in the internal consistency (alpha changed from 0.56 to 0.60).

Table S8
Correlation Coefficients between Variables in Own Communicative Behavior

	2	3	4	5	6	7
Overall						
Own language quantity						
1. Toward children	0.53	-0.06	-0.13	0.04	-0.02	-0.08
2. Toward team members		0.00	0.03	-0.01	0.07	-0.03
Own language quality						
3. Toward children			0.73	0.30	0.36	NA
4. Toward team members				0.26	0.37	0.63
Own paralinguistic cues						
5. Toward children					0.51	0.24
6. Toward team members						0.33
Own language quality						
7. Toward children*						
France						
Own language quantity						
1. Toward children	0.62	0.02	-0.02	0.06	0.04	0.00
2. Toward team members		0.03	0.10	-0.02	0.09	0.02
Own language quality						
3. Toward children			0.67	0.25	0.34	NA
4. Toward team members				0.18	0.29	0.53
Own paralinguistic cues						
5. Toward children					0.52	0.20
6. Toward team members						0.28
Own language quality						
7. Toward children*						
Japan						
Own language quantity						
1. Toward children	0.21	0.15	0.08	0.10	0.03	0.11
2. Toward team members		0.06	0.04	0.04	0.07	-0.04
Own language quality						
3. Toward children			0.66	0.35	0.26	NA
4. Toward team members				0.37	0.38	0.54
Own paralinguistic cues						
5. Toward children					0.48	0.23
6. Toward team members						0.28
Own language quality						
7. Toward children*						

Notes. *The item of 'articulation' (*r*.drop = 0.30) was eliminated, which led to an increase in the internal consistency (alpha changed from 0.56 to 0.60).

Table S9

Comparison of Subscale Scores between Directors and Non-Directors in Each Country

Subscale	French			Japanese		
	Director	Non-director		Director	Non-director	
	Mean (SD)	Mean (SD)	Adj. *p*	Mean (SD)	Mean (SD)	Adj. *p*
Own language quantity						
Toward children	-0.27 (0.46)	-0.24 (0.66)	1.0	-0.01 (0.44)	0.02 (0.34)	1.0
Toward team members	-0.25 (0.56)	-0.27 (0.83)	1.0	-0.19 (0.36)	-0.15 (0.40)	1.0
Own language quality						
Toward children	1.04 (0.59)	1.22 (0.65)	1.0	0.56 (0.39)	0.64 (0.52)	1.0
Toward team members	1.03 (0.58)	1.23 (0.73)	1.0	0.38 (0.58)	0.47 (0.49)	1.0
Own paralinguistic cues						
Toward children	0.75 (0.53)	0.77 (0.67)	1.0	0.60 (0.39)	0.66 (0.39)	1.0
Toward team members	0.59 (0.57)	0.70 (0.82)	1.0	0.06 (0.51)	0.45 (0.57)	0.36
Own ease of exchange with team members	**-0.96 (0.56)**	**-1.31 (0.53)**	**0.0058**	-0.46 (0.44)	-0.69 (0.48)	1.0
Verbal communicative behavior						
In infants	-0.59 (0.62)	-0.66 (0.73)	1.0	-0.36 (0.60)	-0.21 (0.51)	1.0
In toddlers	-0.39 (0.53)	-0.51 (0.64)	1.0	-0.24 (0.52)	-0.13 (0.38)	1.0
In team members	-0.13 (0.43)	-0.14 (0.51)	1.0	-0.13 (0.34)	0.00 (0.31)	1.0
Non-verbal communicative behavior						
In infants	-0.15 (0.47)	-0.06 (0.54)	1.0	-0.14 (0.27)	-0.02 (0.40)	1.0
In toddlers	-0.02 (0.40)	0.08 80.46)	1.0	-0.10 (0.31)	0.00 (0.24)	1.0
Ease of exchange with the educator						
For infants	-0.73 (0.58)	-0.93 (0.62)	1.0	-0.50 (0.55)	-0.58 (0.55)	1.0
For toddlers	**-0.77 (0.47)**	**-1.15 (0.55)**	**< 0.001**	-0.56 (0.45)	-0.54 (0.51)	1.0
For team members	-1.07 (0.60)	-1.37 (0.59)	0.085	-0.41 (0.42)	-0.56 (0.53)	1.0
Own worry about children's development	48.6 (28.8)	40.3 (29.1)	1.0	62.6 (33.0)	41.8 (31.2)	0.90
Own language development knowledge	17.3 (2.5)	16.1 (2.8)	0.21	15.6 (3.0)	14.3 (3.3)	1.0
Own attitude towards mask wearing	2.28 (1.04)	1.70 (1.10)	1.0	2.69 (1.01)	2.58 (0.71)	1.0

Notes. Holm's method was used for the *p*-value adjustment. Significant results were shown in bold.
